# Supplementary material for: Flooding and hydrologic connectivity modulate community assembly in a dynamic river-floodplain ecosystem
Source: PLoS One. 2019 Apr 12;14(4):e0213227. doi: 10.1371/journal.pone.0213227 (PMC6461263; doi:10.1371/journal.pone.0213227)
Supplement: S1 Appendix — (DOCX) [file pone.0213227.s001.docx]

**S1 Appendix. Identification keys used in the study**

*Oligochaeta*

Brinkhurst, R. O. and Jamieson, B. G. M. E. 1971. Aquatic Oligochaeta of the World. Edinburg:860 pp.

Cekanovskaya, O. V., 1962. The aquatic Oligochaete fauna of the USSR. - Opred Faune SSSR 78:411 pp.

Hrabě, S., 1954. Máloštětinatci - Oligochaeta. Klíč zviřeny ČSR Praha 1:287-323.

Hrabě, S., 1981. Vodní máloštětinatci (Oligochaeta) Československa. - Acta Universitatis Carolinae - Biologica 1-2 1979:167 pp.

Kasprzak, K., 1981. Skaposzczety wodne I. Klucze do oznaczania bezkregowcov Polski Warszawa 4:226 pp.

Sperber, C., 1950. A guide for the determination of the European Naididae. - Zool Bidr Upps, 29:78pp.

*Chironomidae*

Wiederholm, T. 1983. Chironomidae of the Holarctic Region. Part. 1: Larvae. - Entomologica Scandinavica Supplement, 19, 457.

Bitušík, P., 2000. Príručka na určovanie lariev pakomárov (Diptera: Chironomidae) Slovenska. Časť I. Buchonomyinae, Diamesinae, Prodiamesinae a Orthocladiinae. Zvolen: Vyd. Tech. Univerzity vo Zvolene. 133 pp.

Andersen, Ti, Cranston P.S., Epler JH, 2013. Chironomidae of the Holarctic Region: Keys and diagnoses - Larvae. Entomological Society, Lund:  571 pp.
