# Supplementary material for: Flooding and hydrologic connectivity modulate community assembly in a dynamic river-floodplain ecosystem
Source: PLoS One. 2019 Apr 12;14(4):e0213227. doi: 10.1371/journal.pone.0213227 (PMC6461263; doi:10.1371/journal.pone.0213227)
Supplement: S1 Table — Degree of hydrologic connectivity and main physico-chemical characteristics of each floodplain waterbody. Density and richness of the invertebrate communities is also shown. (DOCX) [file pone.0213227.s003.docx]

**S1 Table. Main characteristics of the study floodplain**

Degree of hydrologic connectivity and main physico-chemical characteristics of each floodplain waterbody. Density and richness of the invertebrate communities is also shown.
