# Supplementary material for: Flooding and hydrologic connectivity modulate community assembly in a dynamic river-floodplain ecosystem
Source: PLoS One. 2019 Apr 12;14(4):e0213227. doi: 10.1371/journal.pone.0213227 (PMC6461263; doi:10.1371/journal.pone.0213227)
Supplement: S1 Fig — Correlation patterns between observed richness and lateral hydrologic connectivity (%) for each month. A smooth line is included to show the variation in the pattern through time. (DOCX) [file pone.0213227.s005.docx]

**S1 Fig. Richness-connectivity over time**

Correlation patterns between observed richness and lateral hydrologic connectivity (%) for each month. A smooth line is included to show the variation in the pattern through time.
