# Supplementary material for: Flooding and hydrologic connectivity modulate community assembly in a dynamic river-floodplain ecosystem
Source: PLoS One. 2019 Apr 12;14(4):e0213227. doi: 10.1371/journal.pone.0213227 (PMC6461263; doi:10.1371/journal.pone.0213227)
Supplement: S2 Fig — Temporal variation in the composition of each floodplain waterbody as expressed by the first Correspondence Analysis (CA) factorial plane. For each waterbody, the degree of hydrologic connectivity (%) is also shown. Each point represent one month. Decreasing variation (especially along the first axis) with increasing connectivity is evident. (DOCX) [file pone.0213227.s006.docx]

**S2 Fig. Temporal change in faunal composition**

Temporal variation in the composition of each floodplain waterbody as expressed by the first Correspondence Analysis (CA) factorial plane. For each waterbody, the degree of hydrologic connectivity (%) is also shown. Each point represent one month. Decreasing variation (especially along the first axis) with increasing connectivity is evident.
