# Supplementary material for: Flooding and hydrologic connectivity modulate community assembly in a dynamic river-floodplain ecosystem
Source: PLoS One. 2019 Apr 12;14(4):e0213227. doi: 10.1371/journal.pone.0213227 (PMC6461263; doi:10.1371/journal.pone.0213227)

**S3 Fig. Composition changes after floods**

Relationship between hydrologic connectivity and changes in taxonomic composition as expressed by the first Correspondence Analysis (CA) axis scores. Each panel shows changes in CA axis 1 scores between consecutive months during which flooding occurred. No significant correlations were observed, but for clarity a dashed line was included to show the direction of change.


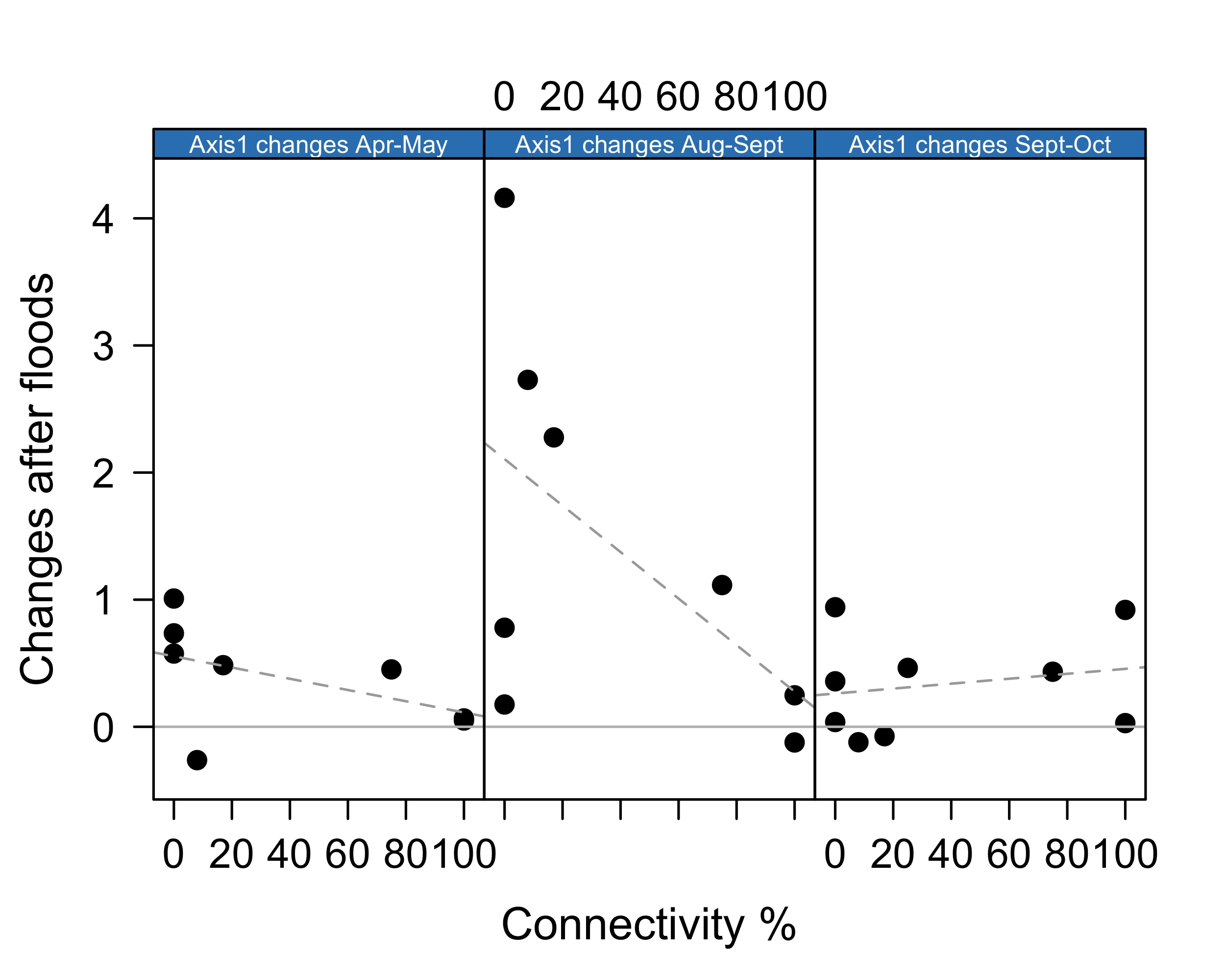

Supplement: S3 Fig — Relationship between hydrologic connectivity and changes in taxonomic composition as expressed by the first Correpondence Analysis (CA) axis scores. Each panel shows changes in CA axis 1 scores between consecutive months during which flooding occurred. No significant correlations were observed, but for clarity a dashed line was included to show the direction of change. (DOCX) [file pone.0213227.s007.docx]
