# Supplementary material for: Flooding and hydrologic connectivity modulate community assembly in a dynamic river-floodplain ecosystem
Source: PLoS One. 2019 Apr 12;14(4):e0213227. doi: 10.1371/journal.pone.0213227 (PMC6461263; doi:10.1371/journal.pone.0213227)
Supplement: S4 Fig — Temporal changes in mean (SE) Euclidean distance among floodplain waterbodies based on the main physico-chemical parameters. Blue vertical lines represent flooding events. There is no consistent indication of a decline in distance (i.e. abiotic homogenisation) with the occurrence of floods. Data from the first and last months (month 4 and 3, respectively) were not available. (DOCX) [file pone.0213227.s008.docx]

**S4 Fig. Temporal changes in abiotic distance between sites**

Temporal changes in mean (SE) Euclidean distance among floodplain waterbodies based on the main physico-chemical parameters. Blue vertical lines represent flooding events. There is no consistent indication of a decline in distance (i.e. abiotic homogenisation) with the occurrence of floods. Data from the first and last months (month 4 and 3, respectively) were not available.
